# Cyclooxygenase-2 regulates TGFβ-induced cancer stemness in triple-negative breast cancer

**DOI:** 10.1038/srep40258

**Published:** 2017-01-05

**Authors:** Jun Tian, Mahmood Y. Hachim, Ibrahim Y. Hachim, Meiou Dai, Chieh Lo, Fatmah Al Raffa, Suhad Ali, Jean Jacques Lebrun

**Affiliations:** 1 Department of Medicine, McGill University Health Center, Cancer Research Program, Montreal, Quebec, H4A 3J1, Canada

## Abstract

Triple negative breast cancer (TNBC), an aggressive subtype of breast cancer, display poor prognosis and exhibit resistance to conventional therapies, partly due to an enrichment in breast cancer stem cells (BCSCs). Here, we investigated the role of the cyclooxygenase-2 (COX-2), a downstream target of TGFβ, in regulating BCSCs in TNBC. Bioinformatics analysis revealed that COX-2 is highly expressed in TNBC and that its expression correlated with poor survival outcome in basal subtype of breast cancer. We also found TGFβ-mediated COX-2 expression to be Smad3-dependent and to be required for BCSC self-renewal and expansion in TNBCs. Knocking down COX-2 expression strikingly blocked TGFβ-induced tumorsphere formation and TGFβ-induced enrichment of the two stem-like cell populations, CD24^low^CD44^high^ and ALDH+ BCSCs. Blocking COX-2 activity, using a pharmacological inhibitor also prevented TGFβ-induced BCSC self-renewal. Moreover, we found COX-2 to be required for TGFβ-induced expression of mesenchymal and basal breast cancer markers. In particular, we found that TGFβ-induced expression of fibronectin plays a central role in TGFβ-mediated breast cancer stemness. Together, our results describe a novel role for COX-2 in mediating the TGFβ effects on BCSC properties and imply that targeting the COX-2 pathway may prove useful for the treatment of TNBC by eliminating BCSCs.

TNBCs account for approximately 10 to 20% of all breast cancers and are characterized by the lack expression of estrogen receptor (ER), progesterone receptor (PR), and human epidermal growth factor receptor 2 (HER2). TNBCs are primarily comprised of an intrinsic molecular subtype of breast cancer, the basal-like subtype. Because of the lack of specific targets for this type of tumors, there are currently no available efficient treatment for TNBC^1^,^2^. The majority of TNBC patients are at high risk of tumor relapse and metastases^3^ and more efforts are clearly needed to better understand this disease and to identify new therapeutic options for these deadly cancers.

Recent studies have suggested that cancer stem cells (CSCs), a small subset of cancer cells which possess stemness properties, are capable of initiating and sustaining tumor growth. CSCs also contribute to tumor recurrence, due to their inherent distinct biological properties, such as resistance to chemotherapy and radiotherapy, evasion of cell death, and quiescence^4^,^5^,^6^,^7^. The first CSCs isolated and characterized in solid tumor was from breast cancer. These breast cancers stem cells (BCSCs) were identified by virtue of their cell surface marker CD24^low^CD44^high^ and aldehyde dehydrogenase (ALDH) enzymatic activity^8^,^9^. Using primary breast xenografts in immunodeficient nonobese diabetic (NOD)/severe combined immunodeficiency (SCID) mice, BCSCs were enriched for their tumor-initiating capacity. When serially transplanted, BCSCs are capable of recapitulating the heterogeneity of the primary breast tumor lesions they are derived from. Interestingly, CD24^low^CD44^high^ and ALDH+ cancer cells represent distinct BCSC populations, although overlapping, they also display distinct proliferative, motile and invasive capacities^10^. Evidence also suggests that poorly differentiated (higher grade) breast cancers contain higher amount of BCSCs and display overexpression of embryonic stem (ES)-associated transcriptional regulators^11^. This ES-like gene signature is associated with poor clinical outcome in breast cancer^12^. Combined with other reports implicating BCSCs in innate resistance to cytotoxic agents, tumor relapse and metastases^13^,^14^, these studies emphasize the needs for a better understanding of cancer stemness.

TGFβ signaling is of central importance for various biological processes, ranging from embryogenesis to cancer pathogenesis. In advanced breast cancer, the TGFβ signaling pathways promote tumor progression by modulating cancer cell epithelial-mesenchymal transition (EMT), invasion, migration and metastasis^15^. Recently, several studies have revealed the role of TGFβ in regulating BCSC activity^16^. Human breast cancer cells undergoing EMT in response to TGFβ have been shown to acquire BCSC features, including increased self-renewing capacity and tumorigenicity as well as resistance to chemotherapy^17^. Furthermore, the TGFβ pathway promotes tumorsphere formation *in vitro* and was found to induce the regenerative capacity of tumor-initiating cells *in vivo* in claudin^low^ subtypes of breast cancer^18^. Chemotherapy treatment of breast tumors was also found to potentiate TGFβ signaling in these cancer cells, further leading to the expansion of chemotherapy-resistant population of BCSCs and tumor recurrence^19^. Despite these accumulating evidence suggesting a central role for TGFβ in regulating BCSC function, the downstream molecular targets that relay and mediate the TGFβ effects remain largely unknown.

We previously identified the COX-2 enzyme as a TGFβ downstream target, involved in the TGFβ-mediated regulation of breast cancer cell migration and invasion in TNBCs^20^. COX-2 catalyzes a key step in the formation of prostaglandins (PGs) and is highly induced at inflammatory sites and during tumor progression^21^. Aberrant COX-2 expression was first reported in colorectal carcinoma and has now been extended to various human cancers, including those of the breast^22^,^23^. In fact, in breast cancer, COX-2 expression has been associated with bad prognosis and tumor progression^24^,^25^. COX-2 was also recently linked to CSCs regulation in the context of bladder and colorectal cancers^26^,^27^. Because of COX-2 involvement in the regulation of CSCs in these tumors and of its broad tumor-promoting functions in breast cancer, we investigated the role of COX-2 downstream of TGFβ-mediated breast cancer stemness.

Bioinformatics analysis using large cohorts of breast cancer patients revealed that COX-2 is highly expressed in TNBC and that its expression significantly correlated with poor survival outcomes in basal subtypes of breast cancer. Notably, we found that silencing COX-2 gene expression in TNBCs impaired TGFβ-induced BCSC self-renewal as well as TGFβ-induced CD24^low^CD44^high^ and ALDH+ stem-like cell populations. We also found that blocking COX-2 enzymatic activity, using a specific pharmacological inhibitor efficiently prevented TGFβ effect on BCSC self-renewal. Moreover, we found COX-2 enzymatic activity and its main metabolite, PGE2 to be required for TGFβ-induced BCSC self-renewal. Finally, we found TGFβ-induced COX-2 expression to increase several mesenchymal and basal breast cancer markers and to promote BCSC properties through fibronectin. Taken together, these data describe a function of COX-2 in BCSCs and provide a new potential therapeutic target for the treatment of TNBC.

## Results

### COX-2 is highly expressed in TNBC and correlates with poor survival outcomes

**Figure 1:**
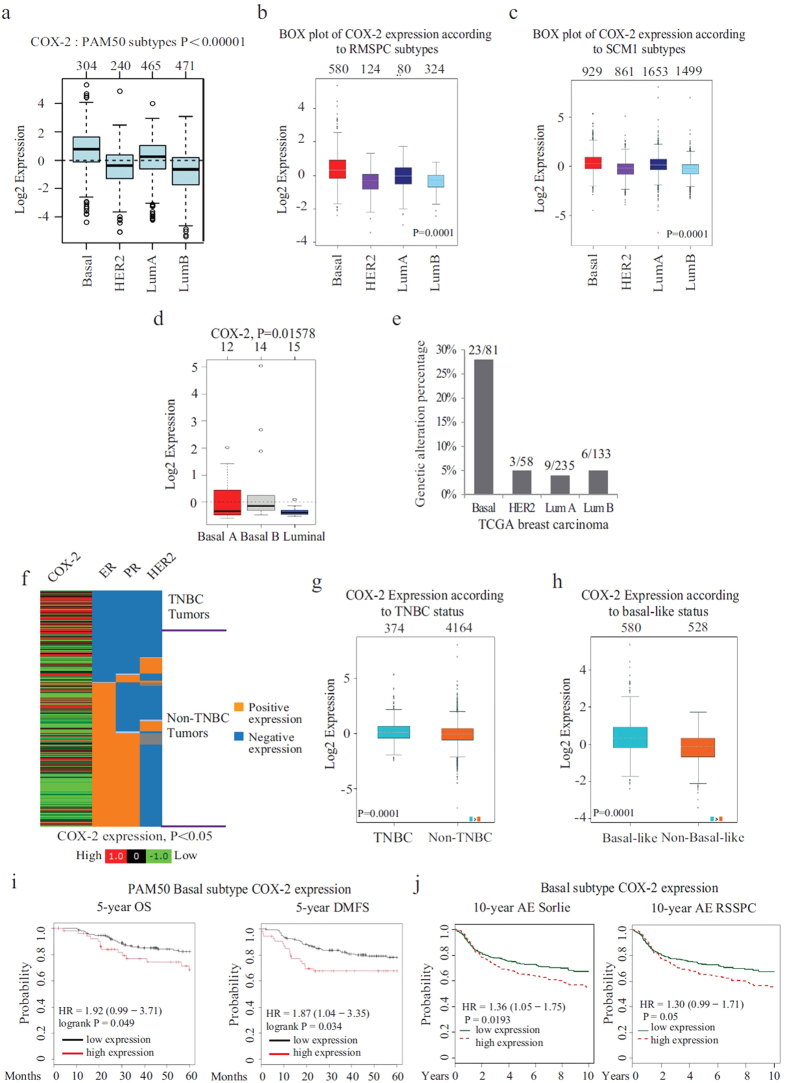
COX-2 is highly expressed in TNBC and correlates with poor survival outcomes. **(a)** Box plot of COX-2 gene expression across PAM50 breast cancer subtypes in GOBO breast cancer dataset. The number of tumor samples is indicated above the box plot. (**b)** Box plot of COX-2 gene expression in different breast cancer subtypes according to RMSPC classification using Breast Cancer Gene-Expression Miner v4.0. The number of tumor samples is indicated above the box plot. (**c)** Box plot of COX-2 gene expression in different breast cancer subtypes according to SCM1 classification using Breast Cancer Gene-Expression Miner v4.0. The number of tumor samples is indicated above the box plot. (**d)** Box plot of COX-2 gene expression in different breast cancer cell lines using GOBO gene set analysis. (**e)** Analysis of COX-2 genetic alterations (amplification, mRNA upregulation or protein upregulation) across various breast cancer subtypes in TCGA breast carcinoma dataset using the cBioPortal. (**f)** Heat map of COX-2 gene expression in 1,215 TNBC and non-TNBC tumors in TCGA breast carcinoma dataset. (**g)** Box plot of COX-2 gene expression in TNBC compared to non-TNBC breast cancer patients using Breast Cancer Gene-Expression Miner v4.0. (**h)** Box plot of COX-2 gene expression in basal-like compared to non-basal like breast cancer patients using Breast Cancer Gene-Expression Miner v4.0. (**i)** Kaplan-Meier survival analysis showing the relationship between COX-2 expression and 5-year OS outcome as well as DMFS outcome in patients who have basal-like tumors (according to PAM50 classification) using Kaplan Meier-plotter database. The survival rates were compared between patients who have low (black) and high (red) levels of COX-2 expression. (**j)** Kaplan-Meier survival analysis showing the relationship between COX-2 expression and AE free survival in patients who have basal-like tumors (according to SORLIE and RSSPC classification) using Breast Cancer Gene-Expression Miner v4.0.

We first analyzed the COX-2 expression levels in various breast cancer subtypes using GOBO (http://co.bmc.lu.se/gobo) online tool in a large dataset (1,881 patients) of breast cancer patients^28^ as well as the Breast Cancer Gene-Expression Miner v4.0 (bc-GenExMiner v4.0) database including 5609 breast cancer patients^29^. Interestingly, analysis of COX-2 expression levels across different molecular subtypes, using different classification methods in both databases, revealed that high COX-2 expression correlated with the most aggressive basal-like breast cancer subtype, compared to HER2 and luminal tumors (Fig. 1a–c and Fig. S1a–c). Further analysis, using 51 different breast cancer cell lines revealed COX-2 expression to be the highest in the basal-b subtype, compared to basal-a and luminal (Fig. 1d). The basal–b subtype is associated with mesenchymal phenotype and stem cell-like features. Moreover, when assessing for COX-2 genetic alterations (gene amplification, mRNA and protein up-regulation) in the various breast cancer subtypes using cBioportal (http://cbioportal.org) online application, we found the basal-like subtype to display the highest rate of COX-2 amplifications, compared to HER2 and luminal subtypes (Fig. 1e). In addition, we further analyzed COX-2 expression in the TCGA breast carcinoma dataset using UCSC Cancer Genomics Browser (https://genome-cancer.ucsc.edu/). Using 1,215 breast cancer tissue samples, we found a significant correlation between high COX-2 expression and TNBC, compared to non-TNBCs (P < 0.05) (Fig. 1f). This results were further confirmed using 4538 breast cancer patient cohort in Breast Cancer Gene-Expression Miner v4.0 database which also revealed a significant higher expression of COX-2 mRNA levels in TNBC tumors compared to non-TNBC tumors (P = 0.0001) (Fig. 1g) and in the more aggressive basal-like subtype of TNBC compared to non-basal-like tumors (Fig. 1h). Together, these data show that COX-2 is highly expressed in basal-like TNBC, suggestive of an association between COX-2 high expression levels and the aggressive behavior of this type of breast tumor.

To start investigating the clinical relevance of COX-2 expression and its association with patient outcome, we further analyzed the association between COX-2 expression and overall survival (OS), distant metastasis free survival (DMFS) and any event (AE) free survival rates, using publically available Kaplan-Meier plotter database as well as the above mentioned Breast Cancer Gene-Expression Miner v4.0. Interestingly, high COX-2 expression was significantly associated with poor patient outcome in basal-like breast cancer tumors represented as shorted OS, DMFS and AE free survival (Hazard Ratio (HR), 1.92; 0.99 to 3.71; p = 0.049); (HR, 1.87; 1.04 to 3.35; p = 0.034), (HR = 1.36; 1.05 to 1.75; P = 0.0193), (HR = 1.30; 0.99 to 1.71; P = 0.05) respectively in these patients (Fig. 1i,j). This correlation was specific to basal-like tumors, as no significant difference was observed in both luminal A and luminal B tumors (Fig. S1d,e). These results indicate the role of COX-2 expression as a marker of poor outcome and higher risk of metastasis in basal-like breast cancer patients.

### TGFβ induction of COX-2 expression in basal-like TNBC is Smad3-dependent

**Figure 2:**
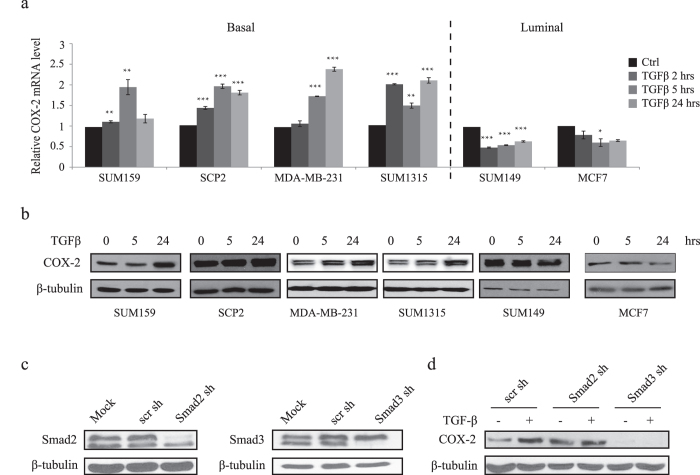
TGFβ/Smad3 signaling upregulates COX-2 expression in basal-like TNBC. **(a** and **b)** The specified breast cancer cells were untreated or treated with TGFβ (200 pM) for the indicated times and mRNA as well as protein levels for COX-2 were measured by real-time qPCR and Western blot. COX-2 proteins were presented in the upper bands in MDA-MB-231, SUM1315 and SUM149. (**c)** SUM159 cells were transfected with scr, Smad2 or Smad3 shRNAs. Total cell lysates were analyzed for Smad2, Smad3 and β-tubulin by Western blot. (**d)** SUM159 cells transfected with the indicated shRNAs were untreated or treated with TGFβ (200 pM) for 24 hours. Western blot was performed using anti-COX-2 and anti-β-tubulin antibodies.

We previously reported that COX-2 expression is regulated by TGFβ/p21 signaling in breast cancer cells and is required for TGFβ-induced breast cancer cell migration and invasion^20^. Thus, to investigate the role and contribution of COX-2 in tumor progression, we next examined the TGFβ effects on COX-2 expression in basal versus luminal breast cancer subtypes. As shown in Fig. 2a, TGFβ induced COX-2 mRNA levels in all basal BC cell lines. Interestingly, TGFβ had an opposite effect in luminal cells and decreased COX-2 mRNA levels (Fig. 2a). The TGFβ effects on COX-2 expression were further analyzed and confirmed at the protein level, using immunoblot analysis (Fig. 2b). These results indicate that TGFβ-mediated COX-2 up-regulation is specific to basal breast cancer.

In the canonical TGFβ signaling pathway, TGFβ interacts with a complex of two serine kinase receptors, which is followed by recruitment and phosphorylation of the receptor-regulated Smad2 and Smad3 transcription factors, which then mediate TGFβ-induced transcriptional response^15^. To then investigate whether increased COX-2 expression is regulated by the canonical TGFβ pathway in TNBC, we generated Smad2 and Smad3 shRNA lentiviral particles to specifically knockdown their respective expression, in basal SUM159 cells (Fig. 2c). Interestingly, while depletion of Smad2 did not affect TGFβ-induced COX-2 expression, Smad3 gene silencing completely abolished the TGFβ response (Fig. 2d). These results indicate that the TGFβ-mediated of COX-2 is dependent on the Smad canonical pathway but Smad3 specific.

### TGFβ/Smad3-induced COX-2 expression is required for BCSC self-renewal in basal-like TNBC

**Figure 3:**
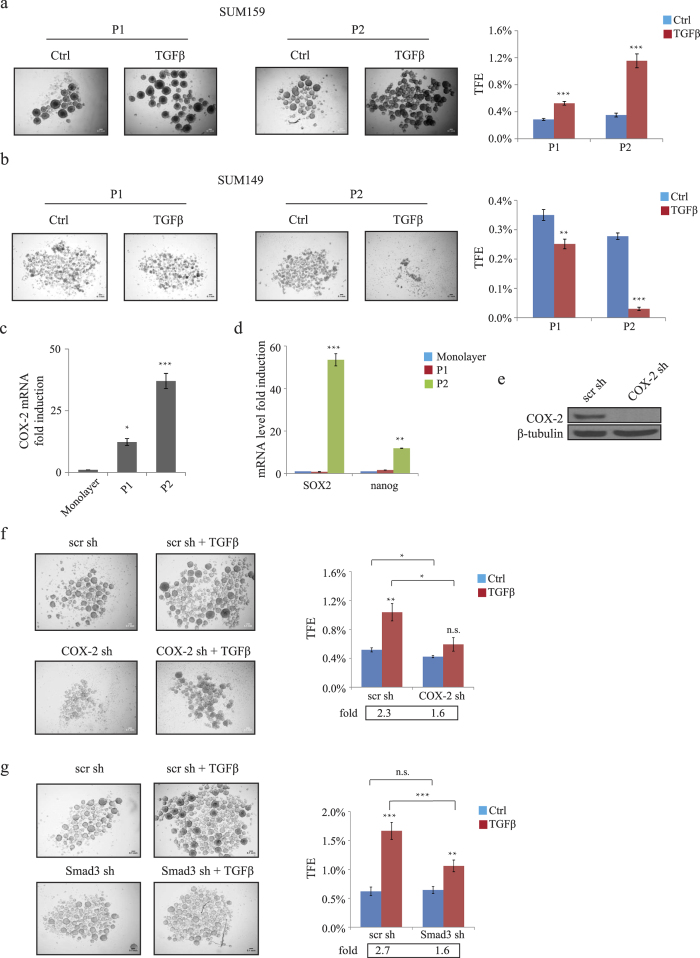
TGFβ/Smad3-induced COX-2 expression regulates tumorsphere formation in basal-like TNBC. (**a** and **b)** Representative images of P1 and P2 tumorspheres from 10,000 cells of SUM159 and SUM149 in the presence or absence of TGFβ (100 pM). Scale bar = 100 μm. The number of tumorspheres (>60 μm diameter) was counted and tumorsphere forming efficiency (TFE) was calculated. (**c)** Total RNA was extracted from adherent SUM159 cells and tumorspheres (P1 and P2). Gene expression of COX-2 was measured by real-time qPCR. (**d)** Gene expression of SOX2 and Nanog in adherent SUM159 cells and tumorspheres (P1 and P2) were measured by real-time qPCR. (**e)** SUM159 cells were transfected with shRNA against COX-2 or a scrambled (scr) shRNA. Cell lysates were then subjected to immunoblotting using COX-2 and β-tubulin antibodies. (**f** and **g)** SUM159 cells transfected with scr, COX-2 or Smad3 shRNAs were subjected to tumorsphere formation assay in the presence or absence of TGFβ (100 pM). Scale bar = 100 μm. The number of tumorspheres (>60 μm diameter) was counted and expressed as TFE.

Interestingly, a recent study showed that TGFβ could promote tumorigenesis through maintenance/stimulation of BCSCs in claudin^low^ breast cancer^18^, even though the underlying mechanisms have not been thoroughly investigated. We thus examined whether TGFβ-mediated COX-2 expression could drive BCSC stemness, using an *in vitro* tumorsphere-forming assay, a standard assay used to assess BCSC self-renewal^30^. Briefly, SUM159 basal breast cancer cells were seeded in serum-free medium supplemented with growth factors under low-attachment conditions in the presence or the absence of TGFβ for one week. Tumorsphere forming efficiency (TFE) was then calculated as the number of generated tumorspheres divided by the number of seeded single cells. First passage tumorspheres (hereafter referred to as P1) were enzymatically dissociated and cultured into secondary tumorspheres (hereafter referred to as P2) to examine the long-term self-renewal capacity of the cancer stem cells. As shown in Fig. 3a,b, TGFβ promoted tumorsphere formation in both P1 and P2 in basal SUM159 cells, while showing the opposite effect in luminal cells (SUM149). Interestingly, no major difference was observed between the TGFβ effects on adherent cell growth in these two cell lines (Fig. S2a), suggesting that TGFβ-mediated tumorsphere growth is not related to its effect on adherent cell growth. The opposing TGFβ effect on tumorsphere formation are consistent with what observed in Fig. 2 and strongly suggest that TGFβ-induced COX-2 expression in basal, but not luminal breast cancer cells leads to TGFβ-induced BCSC self-renewal.

TGFβ treatment of luminal like breast cancer cells has been shown to undergo a phenotypic modification involving their cytoskeleton and adopt a more mesenchymal phenotype to boost invasion^31^. We then asked whether the opposite responses to TGFβ in BCSC self-renewal, observed between luminal and basal breast cancer subtypes were associated with epithelial/mesenchymal phenotypic changes. For this, we examined the TGFβ effects on the expression levels of different mesenchymal markers (fibronectin and n-cadherin) as well as the expression levels of different luminal markers (keratin 18 and mucin 1) in both 2D and 3D sphere cultures of SUM159 and SUM149 cells. As shown in Fig. S2b, TGFβ treatment of the SUM159 cells increased expression of the two mesenchymal markers while decreasing expression the luminal markers in 2D and 3D cultures. Interestingly, similar results were obtained in SUM149 cells, with a noticeable exception on the TGFβ regulation of fibronectin expression in 3D culture, which showed no TGFβ-mediated increased expression (Fig. S2b). These data suggest that fibronectin might play a role in regulating TGFβ-induced BCSC self-renewal in basal but not luminal subtypes of breast cancer.

To evaluate the association of COX-2 induction and BCSC enhancement, we measured COX-2 mRNA expression in monolayer adherent cells as well as P1 and P2 tumorspheres. COX-2 expression was markedly increased following first and second passage of tumorspheres compared to adherent cells (Fig. 3c). As shown in Fig. 3d, P2 also showed significant elevated levels of Nanog and SOX2, two embryonic stem cell (ESC) markers that have been implicated in CSCs function^12^,^32^,^33^. These results suggest that P2 population is enriched in BCSCs with long-term self-renewal capacity and indicate a positive correlation between COX-2 expression and BCSCs enrichment.

To further investigate the role of COX-2 downstream of TGFβ-mediated stemness, COX-2 gene expression was silenced, using RNA interference and the TGFβ effect on formation of tumorsphere was examined as described above. As shown in Fig. 3f, while TGFβ significantly increased tumorsphere numbers, these effects were impaired when COX-2 gene expression was knockdown. Efficiency of the COX-2 knockdown was verified using immunoblot analysis (Fig. 3e). These results clearly indicate that COX-2 is required for TGFβ to promote BCSC self-renewal abilities in basal TNBCs. As shown in Fig. 2d, TGFβ-dependent COX-2 expression is Smad3-dependent. Notably, the effect of knocking down Smad3 had a similar effect to the one observed when COX-2 was silenced (Fig. 3f,g), confirming the importance of COX-2 in mediating the TGFβ effect on BCSC self-renewal.

### COX-2 expression mediates TGFβ-induced CD24^low^CD44^high^ and ALDH+ BCSC populations in basal-like TNBC

In addition to their ability to form tumorspheres in suspension cultures, BCSC numbers can be measured by assessing the cell markers CD44, CD24 and ALDH1. The two BCSC (CD24^low^CD44^high^ and ALDH+) subpopulations have the capacity to seed tumors at limiting dilutions *in vivo*^8^,^9^. CD44 expression has been shown to contribute to the characteristics of BCSCs such as tumor metastasis and drug resistance^34^. ALDH1 expression has also been associated with poor prognosis and decreased overall survival rates in basal-type breast cancer^35^. A recent study has demonstrated that TGFβ increases the number of both CD24^low^CD44^high^ and ALDH+ cells in claudin^low^ breast cancer cells^18^. Having shown that COX-2 is involved in TGFβ/Smad3-induced BCSC self-renewal, this led us to investigate whether COX-2 was required for the induction of CD24^low^CD44^high^ and ALDH+ stem-like cell populations by the TGFβ/Smad3 signaling in basal-like TNBC.

**Figure 4:**
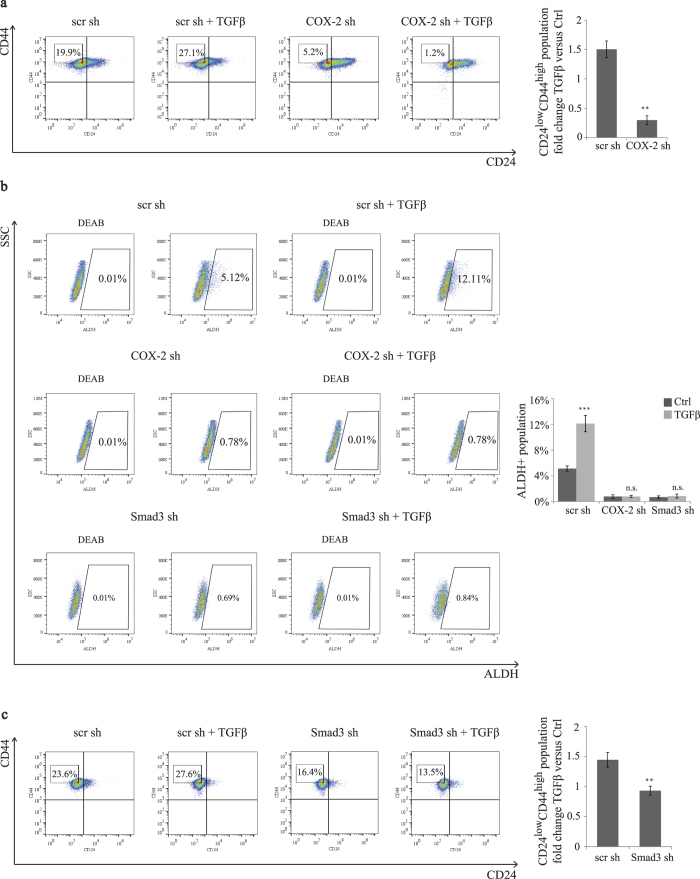
TGFβ/Smad3-induced COX-2 expression regulates the expansion of CD24^low^CD44^high^ and ALDH+ BCSC populations. (**a)** SUM159 cells transfected with scr or COX-2 shRNAs were untreated or treated with TGFβ (200 pM) for 4 days and labeled with an anti-CD44 conjugated to APC antibody and with an anti-CD24 conjugated to PE antibody and analyzed by flow cytometry. Gating was set by control isotype antibodies. The percentage of CD24^low^CD44^high^ populations is indicated. Fold changes of CD24^low^CD44^high^ populations in response to TGFβ were graphed in both scr shRNA and COX-2 shRNA-transfected SUM159 cells. (**b)** SUM159 cells transfected with scr, COX-2 or Smad3 shRNAs were untreated or treated with TGFβ (200 pM) for 4 days and the percentage of ALDH+ cells was analyzed by flow cytometry. The ALDH+ gate was set in reference to control populations incubated with the ALDH inhibitor DEAB. (**c)** SUM159 cells transfected with scr or COX-2 shRNAs were cultured in the presence or absence of TGFβ (200 pM) for 4 days and the percentage as well as fold changes of CD24^low^CD44^high^ populations were assessed and quantified by flow cytometry.

For this, scr shRNA, COX-2 shRNA and Smad3 shRNA-transfected SUM159 were treated with TGFβ for 4 days, after which the proportion of CD24^low^CD44^high^ and ALDH+ populations were examined by flow cytometry analysis. CD24^low^CD44^high^ population was gated based on high 50% of CD44+ population and low 50% of CD24- population, while the ALDH+ population was gated according to the absence of the population in the presence of ALDH inhibitor, DEAB, as previously described^36^. We found TGFβ to increase CD24^low^CD44^high^ proportion from 19.9% to 27.1% and ALDH+ population from 5.12% to 12.11% in scrambled transfected control SUM159 cells (Fig. 4a,b). Interestingly, knocking down COX-2 expression not only markedly reduced the basal levels of CD24^low^CD44^high^ and ALDH+ populations but also blocked the TGFβ effects (Fig. 4a,b). Considering that both of these BCSC populations are capable of forming tumors when transplanted into NOD/SCID mice, these results support the critical role for COX-2 in regulating TGFβ-induced BCSCs expansion. Moreover, we found that depletion of Smad3 using a specific shRNA had similar effects to knocking down COX-2 on TGFβ-induced BCSC subpopulations (Fig. 4b,c), further indicating that TGFβ-mediated COX-2 expression and BCSC expansion are Smad3-dependent. Altogether, these results indicate that COX-2 plays a critical role in regulating the expansion of CD24^low^CD44^high^ and ALDH+ BCSCs downstream of the TGFβ/Smad3 signaling pathway, and further highlight COX-2 as an essential mediator of cancer stemness in basal-like TNBC.

### COX-2 enzymatic activity is required for BCSC self-renewal in basal-like TNBC

**Figure 5:**
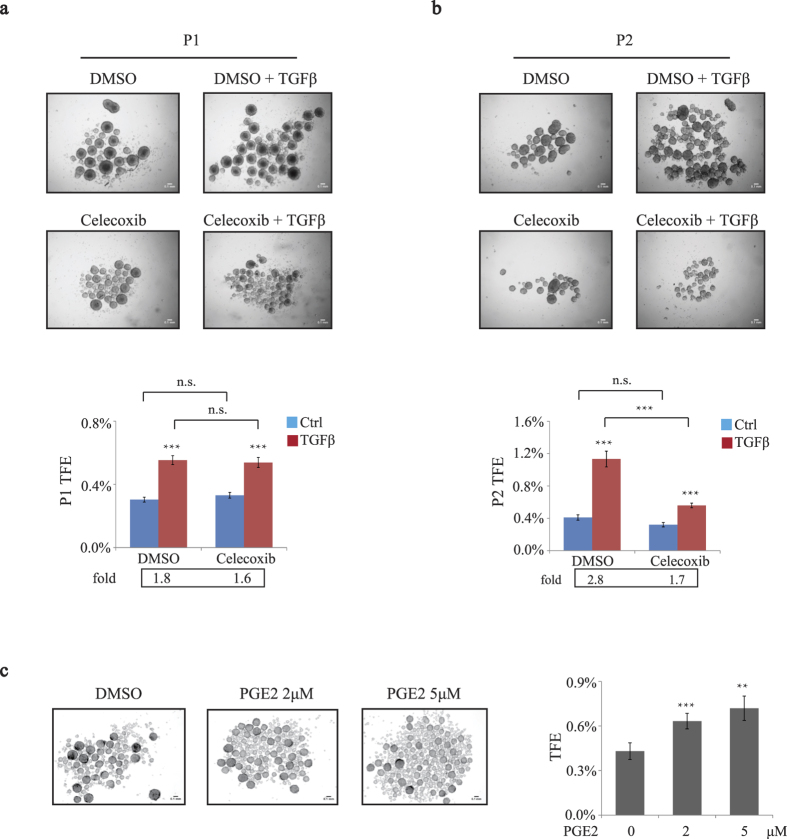
COX-2 enzymatic activity mediates BCSC self-renewal in basal-like TNBC. (**a)** SUM159 cells were treated with or without TGFβ (100 pM) in the presence of either vehicle (DMSO) or 20 μM COX-2 inhibitor (Celecoxib) and subjected to tumorsphere formation assay. The TFE was calculated and graphed. (**b)** The P2 tumorspheres were derived from P1 tumorspheres treated as indicated. The TFE was calculated and graphed. (**c)** Tumorspheres were derived from SUM159 cells treated with DMSO, 2 μM PGE2 or 5 μM PGE2. The number of tumorspheres and TFE were determined.

Previous studies suggested that COX-2 and its metabolite PGE2 contribute to the regulation of CSC expansion in colon and bladder cancers^26^,^27^. COX-2 induction and overexpression have been reported to be associated with high levels of PGE2 in malignant human breast tumors^37^. As a major product of COX-2, PGE2 is known to exert cell-autonomous effects to promote cell proliferation, cell death, tumor invasion and migration as well as landscaping effects to induce angiogenesis in many types of cancer including breast, colon and lung^38^. Furthermore, PGE2 was reported to maintain mammary stem cell state^39^ and to regulate vertebrate hematopoietic stem cell (HSC) homeostasis^40^,^41^. Thus, to assess whether COX-2 enzymatic activity and its metabolite PGE2 were required for TGFβ-induced BCSC self-renewal, we used a potent selective COX-2 pharmacological inhibitor, celecoxib. Although in P1 tumorspheres, celecoxib had little effect on TGFβ-induced tumorsphere formation, it significantly suppressed the TFE of TGFβ-treated basal breast cancer cells in P2 tumorspheres (Fig. 5a,b). These results indicate that COX-2 enzymatic activity is critical to TGFβ–mediated regulation of breast cancer stemness and suggest that COX-2 metabolites may contribute to cancer stemness regulation. This was further investigated by evaluating the role of the main COX-2 enzyme metabolite, PGE2 on tumorsphere formation in breast cancer cells. Interestingly, as shown in Fig. 5c, PGE2 significantly increased the sphere-forming ability in SUM159 cells, in a dose-dependent manner.

Collectively, these results show that the TGFβ-induced self-renewal capacity of BCSCs in breast cancer is dependent of COX-2 expression and activity, and highlight a novel function for COX-2 and its metabolite PGE2 in regulating cancer stemness downstream of TGFβ signaling.

### TGFβ increases the expression of mesenchymal and canonical basal markers through COX-2

**Figure 6:**
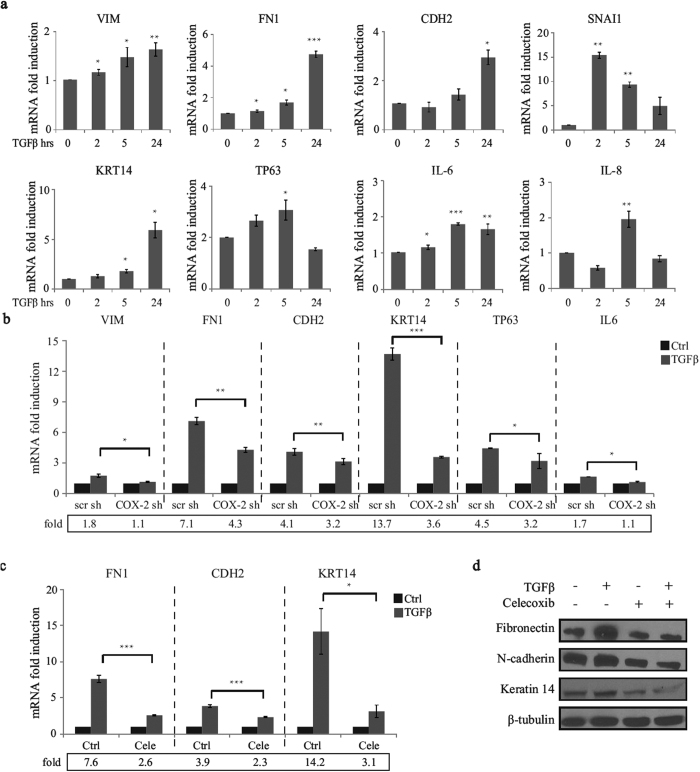
TGFβ increases the expression of mesenchymal and canonical basal markers through COX-2. (**a)** SUM159 cells were untreated or treated with TGFβ (200 pM) for the indicated times and mRNA levels for the indicated genes were measured by real-time qPCR. (**b)** SUM159 cells transfected with scr or COX-2 shRNAs were untreated or treated with TGFβ (200 pM) and mRNA levels for the indicated genes were measured by real-time qPCR. (**c,d)** SUM159 cells were treated with or without TGFβ (200 pM) in the presence of either vehicle (DMSO) or 20 μM COX-2 inhibitor (Celecoxib). Then the mRNA and protein levels of indicated genes were subjected to real-time qPCR and immunoblotting analysis.

Within breast cancers, CSC markers CD24^low^CD44^high^ have been linked with mesenchymal state and aggressive features of breast cancer. This is based on the fact that (1) The gene-expression profiles of CD24^low^CD44^high^–defined CSCs resemble those of basal stem cells and (2) that EMT-associated genes vimentin (VIM), ZEB1, ZEB2, β-catenin (CTNNB1) and matrix metalloproteinase 9 (MMP9) are significantly enriched in the CD24^low^CD44^high^ populations^10^. Moreover, CD24^low^CD44^high^ breast cancer cells have been recently reported to exhibit enhanced invasive properties, thereby contributing to tumor metastasis^42^. Having shown that COX-2 mediated TGFβ-induced CD24^low^CD44^high^ population, this led us to investigate whether COX-2 was required for TGFβ-induced expression of EMT-associated genes and canonical basal markers. We first analyzed the TGFβ effects on EMT-associated genes and basal markers mRNA levels, in a time dependent manner. As shown in Fig. 6a, TGFβ increased expression of all mesenchymal marker tested (vimentin (VIM), fibronectin (FN1), n-cadherin (CDH2), and snail (SNAI1), pro-invasive genes interleukin 6 (IL6), interleukin 8 (IL8) as well as of the expression of the canonical basal markers (keratin 14 (KRT14) and tumor protein p63 (TP63). Interestingly, all of these genes have been newly discovered to be associated with BCSC function/behavior, further supporting the role of TGFβ in promoting cancer cells stemness^10^,^43^,^44^,^45^,^46^,^47^,^48^. Next, to address the involvement of COX-2 in regulating these TGFβ effects, we used COX-2 shRNA and celecoxib to knock down COX-2 expression and to block COX-2 enzyme activity respectively. As shown in Fig. 6b, knocking down of COX-2 expression impaired the TGFβ effects on most of the tested genes (VIM, FN1, CDH2, KRT14, TP63 and IL6). Celecoxib also blocked TGFβ-induced expression of FN1, CDH2 and KRT14, and this was further confirmed by Western blot (Fig. 6c,d). Altogether, these results suggest that COX-2, at least partially, regulates the aggressive mesenchymal state of TNBCs.

### Fibronectin expression is required for TGFβ-induced BCSC self-renewal and expansion of CD24^low^CD44^high^ and ALDH+ cells

**Figure 7:**
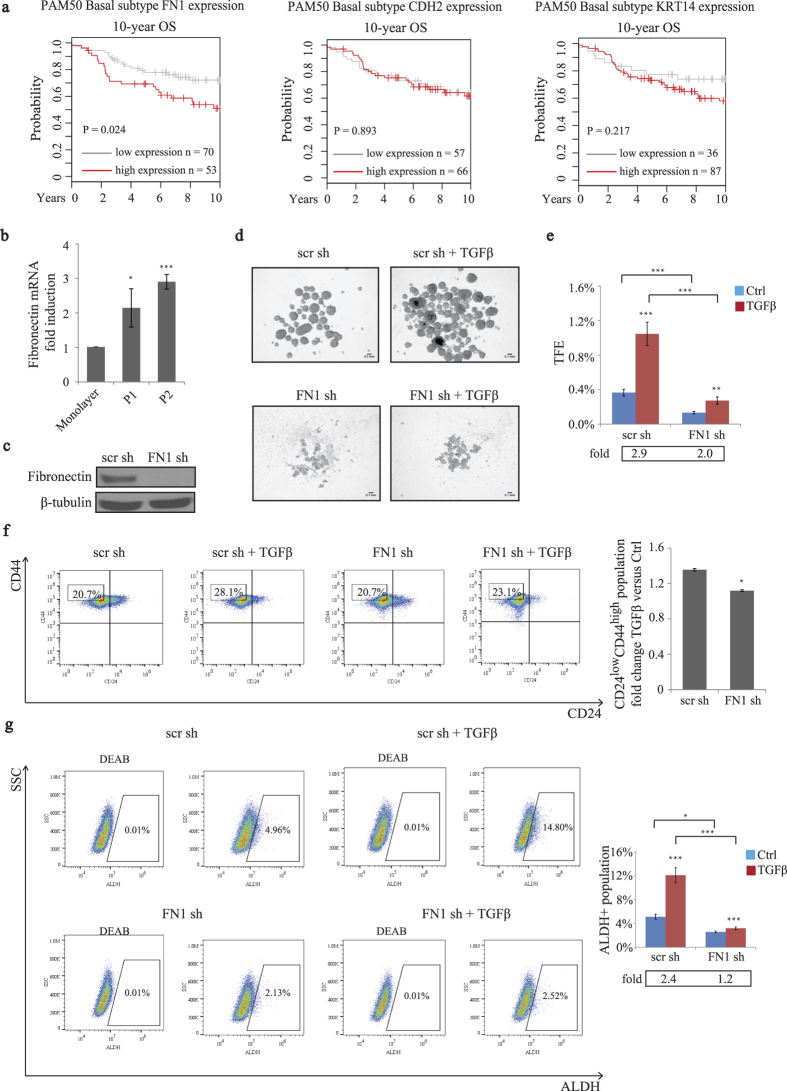
TGFβ/COX-2-induced fibronectin expression regulates cancer stemness in basal-like TNBC. (**a**) Kaplan-Meier survival analysis showing the relationship between the expression of indicated genes and 10-year overall survival outcome in patients who have basal tumors. The survival rates were compared between patients who have low (black) and high (red) levels of genes expression. (**b)** Total RNA was extracted from adherent SUM159 cells and tumorspheres (P1 and P2). Gene expression of fibronectin was measured by real-time qPCR. (**c)** SUM159 cells were transfected with shRNA against FN1 or a scr shRNA. Cell lysates were then subjected to immunoblotting using fibronectin and β-tubulin antibodies. (**d** and **e)** SUM159 cells transfected with scr or FN1 shRNAs were subjected to tumorsphere formation assay in the presence or absence of TGFβ and the TFE was determined. (**f**) SUM159 cells transfected with scr or FN1 shRNAs were untreated or treated with TGFβ and fold changes of CD24^low^CD44^high^ populations in response to TGFβ were graphed. (**g)** Scr shRNA or FN1 shRNA transfected-SUM159 were cultured in the presence or absence of TGFβ. ALDEFluor assay was conducted and percentage of ALDH+ cells was quantified by flow cytometry.

We found that both COX-2 expression and activity concomitantly modulated TGFβ-promoted breast cancer cells stemness and expression of FN1, CDH2 and KRT14. Among these three genes, high fibronectin transcript levels significantly correlated with poor overall survival (OS) outcome in basal breast tumors in a Kaplan-Meier analysis performed in GOBO breast tumor dataset (P < 0.05), whereas high expression of CDH2 and KRT14 did not display any such correlation (Fig. 7a). Given that BCSCs are associated with higher grade and poorer survival outcome in breast cancer, this led us to hypothesize that fibronectin may also be a breast cancer stemness regulator. Fibronectin is a key component of the extracellular matrix and exerts essential functions in the formation of the pre-metastatic niche^49^. To first address the correlation between fibronectin expression and BCSC self-renewal ability, we measured fibronectin mRNA levels in P1 and P2 tumorspheres compared to adherent cells. We found that P1 and P2 tumorspheres displayed higher expression of fibronectin than adherent cells (Fig. 7b), suggesting that fibronectin may regulate BCSC self-renewal capacity. To further assess the contribution of TGFβ-induced fibronectin in mediating breast cancer stemness, the effect of TGFβ on tumorsphere formation was examined in fibronectin depleted SUM159 cells, using a specific FN1 shRNA (Fig. 7c). As shown in Fig. 7d,e, knocking down fibronectin notably impaired both steady-states and TGFβ-mediated BCSC self-renewal capacity. Furthermore, we assessed the role of TGFβ-induced fibronectin in mediating CD24^low^CD44^high^ and ALDH+ populations. For this, scr shRNA and FN1 shRNA-transfected SUM159 cells were treated with TGFβ and the percentage of CD24^low^CD44^high^ and ALDH+ cells were analyzed by flow cytometry. We found that steady-states and expansion of these BCSC populations by TGFβ were impaired in the absence of fibronectin (Fig. 7f,g). All together, these results indicate that, similar to COX-2, fibronectin represents another important player downstream of the TGFβ signaling pathway, which efficiently contributes to sustain BCSC self-renewal *in vitro*.

## Discussion

Treatment options for TNBC patients are extremely limited due to the absence of molecular targets. Despite showing initial responses to chemotherapy, TNBC patients with residual disease have a higher rate of recurrence and a worse prognosis than those with other breast cancer subtypes^50^. Thus, a better understanding of the molecular basis of TNBC and the identification of therapeutic targets for this aggressive type of breast cancer are clearly in need.

COX-2 overexpression has been observed in human breast cancer and associated with mammary carcinogenesis^22^. In this study, we assessed the COX-2 expression levels in different subtypes of breast cancer and the association of COX-2 with patient prognosis. We find COX-2 to display higher expression in TNBC than less aggressive breast cancer subtypes. Elevated COX-2 levels have also been found to correlate with several prognostic parameters of aggressive breast cancer. Consistent with these data, we show that high expression of COX-2 is associated with unfavorable overall and distant metastasis free survival outcomes in basal-like TNBC. Together, our findings indicate the prognostic value of COX-2 expression in basal-like TNBC and highlight the importance to uncover how COX-2 expression contribute to the tumor progression within this type of breast cancer.

The aggressive nature of TNBC is explained in part by the enrichment of BCSCs within the tumor, since BCSCs not only initiate and sustain breast tumor growth, but also play a major role in breast tumor metastasis and resistance to current chemotherapeutic approaches^51^. Thus, targeting and eliminating BCSCs have emerged as interesting therapeutic strategies to eradicate TNBCs. In our study, we establish the linkage between COX-2 and BCSC properties. Indeed, COX-2 expression is elevated in tumorspheres compared to monolayer cells. Knocking down COX-2 expression decreases the TFE as well as selectively reduces the proportion of CD24^low^CD44^high^ and ALDH+ BCSC populations in basal-like TNBC. Consistent with other studies showing the contribution of COX-2 expression on BCSC self-renewal in luminal and HER2+ breast cancer cell lines^52^,^53^, our results further demonstrate the novel function of COX-2 in the regulation of CSCs in TNBC.

Our data clearly indicate that COX-2 does regulate BCSC behavior (self-renewal) and expansion (BCSC sub-population numbers) in basal breast cancer cells, strongly suggesting that COX-2 regulates BCSC activity. However, further experiments, using *in vivo* preclinical animal models would be required to clearly define to what extend the loss of COX-2 expression does affect BCSC activity in promoting tumor formation and/or drug resistance. In addition, it is also possible that the loss of COX-2 gene expression alone is not sufficient to impair BCSC activity *in vivo*. Indeed, other factors/parameters may also be involved in the regulation of these processes. For instance, we recently showed that CDK4 also plays an important role in maintaining BCSC population, activity and drug resistance^36^. It would be interesting to investigate the potential relationship between COX-2 and CDK4 in regulating BCSC behavior and activity.

Here, we show that TGFβ increases BCSC self-renewal in basal-like TNBC, while it exerts an opposite effect on luminal breast cancer cells. This is consistent with a previous report correlating increased self-renewal capacity of claudin^low^ breast cancer cells in response to TGFβ^18^. The dual role of TGFβ in breast cancer cells is well documented, even though not fully understood. Our results showing opposite effects of TGFβ in regulating COX-2 gene expression and BCSC self-renewal in basal versus luminal breast cancer cells could, in part explain why TGFβ promotes cancer progression in more aggressive breast cancer, while it exerts tumor suppressor effects in early carcinomas.

We found the TGFβ effects on COX-2 expression and cancer stemness to be Smad3 dependent in basal breast cancer. Smad2/3 are key mediators of the canonical TGFβ signaling pathway and emerging evidence suggest that Smad2 and Smad3 contribute distinctively to the regulation of TGFβ responses in various contexts^55^,^56^. For instance, Smad3, not Smad2 regulates the TGFβ-induced bone metastasis in breast cancer^57^. Similarly, TGFβ-mediated inhibition of telomerase expression and cell immortalization is Smad3-specific^58^. The present study indicates that the TGFβ effect on cancer stemness is also primarily mediated through Smad3.

We found COX-2 to be required for TGFβ/Smad3-mediated regulation of breast cancer stemness in basal-like TNBCs. These results are in line with the TGFβ effects on tumorsphere formation in distinct subtypes of breast cancer, suggesting that TGFβ-induced COX-2 expression in basal, but not luminal breast cancer cells leads to TGFβ-induced BCSC self-renewal. Moreover, we found the loss of COX-2 expression to markedly prevent TGFβ-induced BCSC self-renewal and expansion of the two BCSC sub-populations (CD24^low^CD44^high^ and ALDH1). These results highlight COX-2 as a primary candidate for targeted therapies to TNBC.

It has been established that selective COX-2 inhibitors reduce breast cancer risk by promoting apoptosis, and by inhibiting cell proliferation and angiogenesis through decreased prostaglandin synthesis^59^,^60^. A clinical study in breast cancer showed that pre-operative celecoxib treatment sets up transcriptional programs supporting anti-tumor activity^61^. Several other trials demonstrated the use of combination of celecoxib and aromatase inhibitors in the neoadjuvant treatment is effective in reducing breast tumor size and area^62^,^63^,^64^. Given the tumor suppressive function of COX-2 inhibitors in breast cancer, combined with the implications of COX-2 enzymatic activity in CSCs in other types of cancer, we tested the use of COX-2 inhibitor in BCSC self-renewal. Interestingly, celecoxib not only impairs the growth of tumorsphere under basal conditions but also potently blocks the TGFβ-dependent effect on tumorsphere formation, similar to that observed when knocking down COX-2 expression. Moreover, PGE2 significantly increases the TFE in basal breast cancer cells. This indicate that both COX-2 expression and enzymatic activity are important for BCSC maintenance and that the beneficial effects of COX-2 inhibitors on breast tumor recurrence and mortality are likely related to decreased BCSC self-renewal capacity.

The presence of BCSCs has also been linked to mesenchymal state and aggressive features of breast cancer. TGFβ has been reported to generate and maintain mesenchymal tumor cells with BCSC characteristics. We showed that TGFβ upregulates the expression of multiple mesenchymal markers and canonical basal markers in TNBC cells. Among these genes, the expression levels of TGFβ-induced fibronectin, n-cadherin and keratin14 are blocked upon suppression of COX-2 expression and enzymatic activity, suggesting COX-2, at least partially, acts as a regulator for aggressive mesenchymal state of TNBC.

Considering the significant correlation between high fibronectin levels and poor overall survival (OS) outcome in basal breast tumors, this suggested that fibronectin may represent a critical downstream regulator of the TGFβ/Smad3/COX-2 effects on cancer stemness. Interestingly, knocking down fibronectin expression strongly inhibits TGFβ-induced BCSC self-renewal and generation of CD24^low^CD44^high^ and ALDH+ BCSC populations. These also highlight fibronectin as an interesting prospective new molecular target for TNBC treatments.

Taken together, this study demonstrates TGFβ-induced COX-2 and fibronectin function as a key regulator axis for BCSC generation and self-renewal in TNBC. It also reveals a potential prognostic value for COX-2 in basal-like breast cancer, as reflected by the correlation between high COX-2 expression levels with poor outcomes. Therapeutic targeting of this pathway might be an attractive strategy for TNBC patients through elimination of BCSCs.

## Methods

All experimental protocols and procedures were performed in accordance to McGill University regulations. All experimental protocols and procedures were approved by McGill University.

### Cell culture

Human breast cancer cell lines SUM159PT and SUM149PT were cultured in F-12 HAM’S serum (Wisent) supplemented with 5% fetal bovine serum (FBS), 5 μg/ml insulin (Sigma-Aldrich, St. Louis, MO, USA), and 1 μg/ml hydrocortisone (Sigma). SCP2, MDA-MB-231 and MCF7 cells were grown in DMEM (Wisent) with 10% FBS and 2 mM L-glutamine. SUM1315MO_2_ was grown in F-12 HAM’S serum (Wisent) containing 5% FBS, 5 μg/ml insulin (Sigma), 10 ng/ml epidermal growth factor (EGF) (Sigma). All cell lines were cultured at 37 °C with 5% CO_2_.

### Lentiviral infection

HEK293T cells were cultured to 90% confluence and transfected with scrambled, Smad3, COX-2 and FN-1 shRNA as well as packaging plasmids (psPAX2 and pMD2.G). Transfections were carried out using bPEI (Sigma) and Opti-MEM^®^ (Invitrogen). After 36–48 hours post-transfection, cell culture medium with lentiviruses were collected. SUM159 cells were plated to 70–80% confluence and infected with lentivirus using 8 μg/ml polybrene. Cells were selected with 5 μg/ml puromycin for 3 days post infection.

### RNA isolation and qRT-PCR for mRNA detection

Total RNA was isolated from cells using TRIzol reagents (Invitrogen) according to manufacturer’s protocol. RNA samples were reverse-transcribed using M-MLV reverse transcriptase (Invitrogen) and random hexamers, as per the manufacturer’s instructions. Subsequently, real-time PCRs were performed using SsoFast^TM^ EvaGreen^®^ Supermix (Bio-Rad) with GAPDH as an internal control. Conditions for qPCR were as follows: 95 °C for 30 s, 40 cycles (95 °C for 5 s and 60 °C for 20 s). Primer sequences were as follows: *PTGS2* forward primer, AGCTTTCACCAACGGGCTGGG; reverse primer, AAGACCTCCTGCCCCACAGCAA; *SOX2* forward primer, TGGACAGTTACGCGCACAT; reverse primer, CGAGTAGGACATGCTGTACGT; *NANOG* forward primer, CATGAGTGTGGATCCAGCTTC; reverse primer, CCTGAATAAGCAGATCCATGG; *FN1* forward primer, CCATCGCAAACCGCTGCCAT; reverse primer, AACACTTCTCAGCTATGGGCTT; *CDH2* forward primer, ATCCTACTGGACGGTTCG; reverse primer, TTGGCTAATGGCACTTGA; *VIM* forward primer, CCAGAGGGAGTGAATCCAGATTA; reverse primer, GAACGCCAGATGCGTGAAATG; *SNAI1* forward primer, TCGGAAGCCTAACTACAGCGA, reverse primer, AGATGAGCATTGGCAGCGAG; *KRT14* forward primer, AGAACCTCAATGACCGCCTG; reverse primer, GTCCACTGTGGCTGTGAGAA; *TP63* forward primer, AACGGTGATGGTACGAAGCG; reverse primer, CATAAGTCTCACGGCCCCTC; *IL6* forward primer, CTCCCCTCCAGGAGCCCAGC; reverse primer, GCAGGGAAGGCAGCAGGCAA; *IL8* forward primer, GCAGAGGCCACCTGGATTGTGC; reverse primer, TGGCATGTTGCAGGCTCCTCAGAA; *KRT18* forward primer, GATCATCGAGGACCTGAGGG; reverse primer, GTGTCATCAATGACCTTGCGG; *MUC1* forward primer, ACAGCTACCACAGCCCCTA; reverse primer, TTGGAGAGGCCCAGAAAACC; GAPDH forward primer, GCCTCAAGATCATCAGCAGCAATGCCT; reverse primer, TGTGGTCATGAGTCCTTCCACGAT.

### Western blot analysis

Cells were lysed in cold lysate buffer containing 10 mM Tris-HCL, pH 7.5, 5 mM EDTA, 150 mM NaCl, 30 mM sodium pyrophosphate, 50 mM sodium fluoride, 1 mM sodium orthovanadate, 1% Triton X-100 and protease inhibitors (1 mM phenylmethylsulfonyl fluoride, 10 μg/ml leupeptin hydrochloride, 10 μg/ml aprotinin and 10 μg/ml pepstatin A). Total protein lysates were quantified, and lysates containing 80 μg of total protein were separated by SDS-PAGE, transferred to nitrocellulose, and subjected to Western blot analysis. The primary antibodies used for Western blot analysis were rabbit polyclonal Smad2/3 antibody (Santa Cruz Biotechnology), rabbit monoclonal COX-2 antibody (Cell Signaling), mouse monoclonal fibronectin antibody (Santa Cruz Biotechnology), mouse monoclonal N-Cadherin antibody (BD Biosciences) and keratin 14 (abcam).

### Tumorsphere formation assay

Cells were seeded at 10,000 cells per well in ultra low-attachment 24-well plate (Corning), and then cultured in serum-free HAM’S F12 medium supplemented with 10 ng/ml EGF, 10 ng/ml bFGF and 1 × B27 (Invitrogen). For the treatment, 100 pM TGFβ or 20 μM celecoxib was added at the moment when the cells were plated. The plate was incubated at 37 °C with 5% CO_2_ for 7 days, without touching the plate. To generate secondary tumorspheres, first passage tumorspheres were enzymatically dissociated and plated at 10,000 cells per well into fresh ultra low-attachment 24-well plate. Tumorspheres from both passages that are 60μm or larger in size were counted. Tumorsphere-forming efficiency (TFE) was calculated using the following equation: TFE (%) = (# of spheres)/(# of cells plated) × 100%.

### Flow cytometry analysis

Monolayer cells were dissociated, filtered through 40 μm cell strainer and counted. 250,000 cells were washed with PBS and then resuspended in FACS buffer (1X PBS, 1% bovine serum albumin [BSA]). Antibodies (PE-conjugated anti-CD24 and APC-conjugated anti-CD44) were added and incubated for 30 minutes on ice. As negative controls for flow cytometry analysis, we used isotype-match conjugated non-immune antibodies. All antibodies were from BD Biosciences. Samples were then washed and analyzed using Accuri C6 flow cytometer (BD Biosciences) and Flowjo software (Tree Star Inc.).

Aldehyde dehydrogenase (ALDH) enzyme activity was measured using an ALDEFLUOR^TM^ Kit (Stemcell Technologies) according to the manufacturer’s protocol. 1 × 10^6^/ml cells were suspended in ALDH assay buffer. 5 μl ALDH substrate (Bodipy-Aminoacetaldehyde) was added and incubated for 45 minutes at 37 °C. For the negative control, the cells were suspended in ALDH buffer containing substrate in the presence of diethylaminobenzaldehyde (DEAB). The ALDH+ cells were detected in the green fluorescent channel of Accuri C6 flow cytometer and the data was analyzed using Flowjo software.

### Cell Viability Assay

SUM159 (10,000 cells per well) and SUM149 cells (20,000 cells per well) were plated into 96-well plates and cultured in F12 medium supplemented with 2% FBS for 48 hours. For the treatment, 100 pM TGFβ was added at the moment when the cells were plated. After two days, cells were incubated with 25 μl 5 mg/ml 3-(4,5-dimethylthiazol-2-yl)-2,5-diphenyltetrazolium bromide (MTT; thiazolyl blue tetrazolium bromide) solution for 2 hours. Dimethyl sulfoxide (DMSO) (200 μl per well) was added and mixed well, then subjected to absorption reading at 570 nm.

### Data mining

Breast Cancer Gene-Expression Miner Version 4.0 (bc-GenExMiner 4.0), GOBO and UCSC Cancer Genomics Browser (https://genome-cancer.ucsc.edu/) online tools was used to evaluate the expression levels of COX-2 in different molecular subtypes. Breast Cancer Gene-Expression Miner include data of 5609 breast cancer patients and allow molecular subtype classification using different classification methods including three single sample predictors (SSPs) methods and three subtype clustering models (SCMs). GOBO database, which is another independent publically available database including 1881 breast cancer patients was also used to evaluate COX-2 mRNA expression in different molecular subtypes.

In addition, Kaplan-Meier plotter database and the prognosis gene expression analysis tool of bc-GenExMiner 4.0 was used to evaluate the association between COX-2 mRNA and patient outcome represented as Overall survival (OS), distant metastasis free survival (DMFS) and any event free survival. cBioportal (http://cbioportal.org) online application was also used to evaluate COX-2 genetic alterations in the various breast cancer subtypes.

### Statistical analyses

Data were expressed as the mean ± SEM of three or more individual experiments. Student’s *t*-test was used to evaluate differences between groups.

## Additional Information

**How to cite this article**: Tian, J. *et al*. Cyclooxygenase-2 regulates TGFβ-induced cancer stemness in triple-negative breast cancer. *Sci. Rep.* **7**, 40258; doi: 10.1038/srep40258 (2017).

**Publisher's note:** Springer Nature remains neutral with regard to jurisdictional claims in published maps and institutional affiliations.

## Supplementary Material

Supplementary Information

## References

[b1] Foulkes W. D., Smith I. E. & Reis-Filho J. S. Triple-negative breast cancer. The New England journal of medicine 363, 1938–1948, doi: 10.1056/NEJMra1001389 (2010). 21067385

[b2] Schneider B. P. . Triple-negative breast cancer: risk factors to potential targets. Clinical cancer research: an official journal of the American Association for Cancer Research 14, 8010–8018, doi: 10.1158/1078-0432.ccr-08-1208 (2008). 19088017

[b3] Andre F. & Zielinski C. C. Optimal strategies for the treatment of metastatic triple-negative breast cancer with currently approved agents. Annals of oncology : official journal of the European Society for Medical Oncology/ESMO 23 Suppl 6, vi46-51, doi: 10.1093/annonc/mds195 (2012). 23012302

[b4] Magee J. A., Piskounova E. & Morrison S. J. Cancer stem cells: impact, heterogeneity, and uncertainty. Cancer cell 21, 283–296, doi: 10.1016/j.ccr.2012.03.003 (2012). 22439924 PMC4504432

[b5] Oskarsson T., Batlle E. & Massague J. Metastatic stem cells: sources, niches, and vital pathways. Cell stem cell 14, 306–321, doi: 10.1016/j.stem.2014.02.002 (2014). 24607405 PMC3998185

[b6] Visvader J. E. & Lindeman G. J. Cancer stem cells: current status and evolving complexities. Cell stem cell 10, 717–728, doi: 10.1016/j.stem.2012.05.007 (2012). 22704512

[b7] Allan A. L., Vantyghem S. A., Tuck A. B. & Chambers A. F. Tumor dormancy and cancer stem cells: implications for the biology and treatment of breast cancer metastasis. Breast disease 26, 87–98 (2006). 17473368 10.3233/bd-2007-26108

[b8] Al-Hajj M., Wicha M. S., Benito-Hernandez A., Morrison S. J. & Clarke M. F. Prospective identification of tumorigenic breast cancer cells. Proceedings of the National Academy of Sciences of the United States of America 100, 3983–3988, doi: 10.1073/pnas.0530291100 (2003). 12629218 PMC153034

[b9] Ginestier C. . ALDH1 is a marker of normal and malignant human mammary stem cells and a predictor of poor clinical outcome. Cell stem cell 1, 555–567, doi: 10.1016/j.stem.2007.08.014 (2007). 18371393 PMC2423808

[b10] Liu S. . Breast cancer stem cells transition between epithelial and mesenchymal states reflective of their normal counterparts. Stem cell reports 2, 78–91, doi: 10.1016/j.stemcr.2013.11.009 (2014). 24511467 PMC3916760

[b11] Pece S. . Biological and molecular heterogeneity of breast cancers correlates with their cancer stem cell content. Cell 140, 62–73, doi: 10.1016/j.cell.2009.12.007 (2010). 20074520

[b12] Ben-Porath I. . An embryonic stem cell-like gene expression signature in poorly differentiated aggressive human tumors. Nature genetics 40, 499–507, doi: 10.1038/ng.127 (2008). 18443585 PMC2912221

[b13] Takebe N., Warren R. Q. & Ivy S. P. Breast cancer growth and metastasis: interplay between cancer stem cells, embryonic signaling pathways and epithelial-to-mesenchymal transition. Breast cancer research : BCR 13, 211, doi: 10.1186/bcr2876 (2011). 21672282 PMC3218933

[b14] Pinto C. A., Widodo E., Waltham M. & Thompson E. W. Breast cancer stem cells and epithelial mesenchymal plasticity - Implications for chemoresistance. Cancer letters 341, 56–62, doi: 10.1016/j.canlet.2013.06.003 (2013). 23830804

[b15] Lebrun J.-J. The Dual Role of TGFβin Human Cancer: From Tumor Suppression to Cancer Metastasis. ISRN Molecular Biology 2012, 1–28, doi: 10.5402/2012/381428 (2012). PMC4899619 27340590

[b16] Scheel C. . Paracrine and autocrine signals induce and maintain mesenchymal and stem cell states in the breast. Cell 145, 926–940, doi: 10.1016/j.cell.2011.04.029 (2011). 21663795 PMC3930331

[b17] Asiedu M. K., Ingle J. N., Behrens M. D., Radisky D. C. & Knutson K. L. TGFbeta/TNF(alpha)-mediated epithelial-mesenchymal transition generates breast cancer stem cells with a claudin-low phenotype. Cancer research 71, 4707–4719, doi: 10.1158/0008-5472.can-10-4554 (2011). 21555371 PMC3129359

[b18] Bruna A. . TGFbeta induces the formation of tumour-initiating cells in claudinlow breast cancer. Nature communications 3, 1055, doi: 10.1038/ncomms2039 (2012). 22968701

[b19] Bhola N. E. . TGF-beta inhibition enhances chemotherapy action against triple-negative breast cancer. The Journal of clinical investigation 123, 1348–1358, doi: 10.1172/jci65416 (2013). 23391723 PMC3582135

[b20] Dai M. . A novel function for p21Cip1 and acetyltransferase p/CAF as critical transcriptional regulators of TGFbeta-mediated breast cancer cell migration and invasion. Breast Cancer Res 14, R127, doi: 10.1186/bcr3322 (2012). 22995475 PMC4053104

[b21] Wang D. & Dubois R. N. Eicosanoids and cancer. Nature reviews. Cancer 10, 181–193, doi: 10.1038/nrc2809 (2010). 20168319 PMC2898136

[b22] Hoellen F. . Impact of cyclooxygenase-2 in breast cancer. Anticancer research 31, 4359–4367 (2011). 22199301

[b23] Yoshimura N. . Expression of cyclooxygenase-1 and -2 in human breast cancer. Surgery today 33, 805–811, doi: 10.1007/s00595-003-2606-3 (2003). 14605950

[b24] Bocca C. . Expression of Cox-2 in human breast cancer cells as a critical determinant of epithelial-to-mesenchymal transition and invasiveness. Expert opinion on therapeutic targets 18, 121–135, doi: 10.1517/14728222.2014.860447 (2014). 24325753

[b25] Ristimaki A. . Prognostic significance of elevated cyclooxygenase-2 expression in breast cancer. Cancer research 62, 632–635 (2002). 11830510

[b26] Kurtova A. V. . Blocking PGE2-induced tumour repopulation abrogates bladder cancer chemoresistance. Nature 517, 209–213, doi: 10.1038/nature14034 (2015). 25470039 PMC4465385

[b27] Ren J. S., Li Q., Guan P., Dai M. & Yang L. [Estimation and prediction for incidence, mortality and prevalence of common gastrointestinal tract cancers in China, in 2008]. Zhonghua Liu Xing Bing Xue Za Zhi 33, 1052–1055 (2012). 23290850

[b28] Ringner M., Fredlund E., Hakkinen J., Borg A. & Staaf J. GOBO: gene expression-based outcome for breast cancer online. PloS one 6, e17911, doi: 10.1371/journal.pone.0017911 (2011). 21445301 PMC3061871

[b29] Jezequel P. . bc-GenExMiner: an easy-to-use online platform for gene prognostic analyses in breast cancer. Breast cancer research and treatment 131, 765–775, doi: 10.1007/s10549-011-1457-7 (2012). 21452023

[b30] Ponti D. . Isolation and *in vitro* propagation of tumorigenic breast cancer cells with stem/progenitor cell properties. Cancer research 65, 5506–5511, doi: 10.1158/0008-5472.can-05-0626 (2005). 15994920

[b31] Cufi S. . Metformin against TGFbeta-induced epithelial-to-mesenchymal transition (EMT): from cancer stem cells to aging-associated fibrosis. Cell cycle (Georgetown, Tex.) 9, 4461–4468, doi: 10.4161/cc.9.22.14048 (2010). 21088486

[b32] Iv Santaliz-Ruiz L. E., Xie X., Old M., Teknos T. N. & Pan Q. Emerging role of nanog in tumorigenesis and cancer stem cells. International journal of cancer. Journal international du cancer 135, 2741–2748, doi: 10.1002/ijc.28690 (2014). 24375318 PMC4065638

[b33] Leis O. . Sox2 expression in breast tumours and activation in breast cancer stem cells. Oncogene 31, 1354–1365, doi: 10.1038/onc.2011.338 (2012). 21822303

[b34] Bourguignon L. Y. Hyaluronan-mediated CD44 activation of RhoGTPase signaling and cytoskeleton function promotes tumor progression. Seminars in cancer biology 18, 251–259, doi: 10.1016/j.semcancer.2008.03.007 (2008). 18450475 PMC2505114

[b35] Charafe-Jauffret E. . Breast cancer cell lines contain functional cancer stem cells with metastatic capacity and a distinct molecular signature. Cancer research 69, 1302–1313, doi: 10.1158/0008-5472.can-08-2741 (2009). 19190339 PMC2819227

[b36] Dai M. . CDK4 regulates cancer stemness and is a novel therapeutic target for triple-negative breast cancer. Sci Rep 6, 35383, doi: 10.1038/srep35383 (2016). 27759034 PMC5069501

[b37] Sobolewski C., Cerella C., Dicato M., Ghibelli L. & Diederich M. The role of cyclooxygenase-2 in cell proliferation and cell death in human malignancies. International journal of cell biology 2010, 215158, doi: 10.1155/2010/215158 (2010). 20339581 PMC2841246

[b38] Gupta R. A. & Dubois R. N. Colorectal cancer prevention and treatment by inhibition of cyclooxygenase-2. Nature reviews. Cancer 1, 11–21, doi: 10.1038/35094017 (2001). 11900248

[b39] Lin M. C. . PGE2 /EP4 Signaling Controls the Transfer of the Mammary Stem Cell State by Lipid Rafts in Extracellular Vesicles. Stem cells (Dayton, Ohio), doi: 10.1002/stem.2476 (2016). 27506158

[b40] Goessling W. . Genetic interaction of PGE2 and Wnt signaling regulates developmental specification of stem cells and regeneration. Cell 136, 1136–1147, doi: 10.1016/j.cell.2009.01.015 (2009). 19303855 PMC2692708

[b41] North T. E. . Prostaglandin E2 regulates vertebrate haematopoietic stem cell homeostasis. Nature 447, 1007–1011, doi: 10.1038/nature05883 (2007). 17581586 PMC2775137

[b42] Sheridan C. . CD44+/CD24− breast cancer cells exhibit enhanced invasive properties: an early step necessary for metastasis. Breast cancer research: BCR 8, R59, doi: 10.1186/bcr1610 (2006). 17062128 PMC1779499

[b43] Qian X. . N-cadherin/FGFR promotes metastasis through epithelial-to-mesenchymal transition and stem/progenitor cell-like properties. Oncogene 33, 3411–3421, doi: 10.1038/onc.2013.310 (2014). 23975425 PMC4051865

[b44] Kim S. Y. . Role of the IL-6-JAK1-STAT3-Oct-4 pathway in the conversion of non-stem cancer cells into cancer stem-like cells. Cellular signalling 25, 961–969, doi: 10.1016/j.cellsig.2013.01.007 (2013). 23333246 PMC3595341

[b45] Singh J. K., Simoes B. M., Howell S. J., Farnie G. & Clarke R. B. Recent advances reveal IL-8 signaling as a potential key to targeting breast cancer stem cells. Breast cancer research: BCR 15, 210, doi: 10.1186/bcr3436 (2013). 24041156 PMC3978717

[b46] Kanojia D. . Proteomic profiling of cancer stem cells derived from primary tumors of HER2/Neu transgenic mice. Proteomics 12, 3407–3415, doi: 10.1002/pmic.201200103 (2012). 22997041

[b47] Memmi E. M. . p63 Sustains self-renewal of mammary cancer stem cells through regulation of Sonic Hedgehog signaling. Proceedings of the National Academy of Sciences of the United States of America 112, 3499–3504, doi: 10.1073/pnas.1500762112 (2015). 25739959 PMC4372004

[b48] Ye X. . Distinct EMT programs control normal mammary stem cells and tumour-initiating cells. Nature 525, 256–260, doi: 10.1038/nature14897 (2015). 26331542 PMC4764075

[b49] Lu P., Weaver V. M. & Werb Z. The extracellular matrix: a dynamic niche in cancer progression. The Journal of cell biology 196, 395–406, doi: 10.1083/jcb.201102147 (2012). 22351925 PMC3283993

[b50] Carey L. A. . The triple negative paradox: primary tumor chemosensitivity of breast cancer subtypes. Clinical cancer research: an official journal of the American Association for Cancer Research 13, 2329–2334, doi: 10.1158/1078-0432.ccr-06-1109 (2007). 17438091

[b51] Wicha M. S., Liu S. & Dontu G. Cancer stem cells: an old idea–a paradigm shift. Cancer research 66, 1883–1890; discussion 1895–1886, doi: 10.1158/0008-5472.can-05-3153 (2006). 16488983

[b52] Majumder M. . COX-2 Induces Breast Cancer Stem Cells via EP4/PI3K/AKT/NOTCH/WNT Axis. Stem cells (Dayton, Ohio), doi: 10.1002/stem.2426 (2016). 27301070

[b53] Singh B. . Role of COX-2 in tumorospheres derived from a breast cancer cell line. The Journal of surgical research 168, e39–49, doi: 10.1016/j.jss.2010.03.003 (2011). 20462604 PMC2921551

[b54] Lebrun J. J. The Dual Role of TGFbeta in Human Cancer: From Tumor Suppression to Cancer Metastasis. ISRN Mol Biol 2012, 381428, doi: 10.5402/2012/381428 (2012). 27340590 PMC4899619

[b55] Brown K. A., Pietenpol J. A. & Moses H. L. A tale of two proteins: differential roles and regulation of Smad2 and Smad3 in TGF-beta signaling. Journal of cellular biochemistry 101, 9–33, doi: 10.1002/jcb.21255 (2007). 17340614

[b56] Kretschmer A. . Differential regulation of TGF-beta signaling through Smad2, Smad3 and Smad4. Oncogene 22, 6748–6763, doi: 10.1038/sj.onc.1206791 (2003). 14555988

[b57] Petersen M. . Smad2 and Smad3 have opposing roles in breast cancer bone metastasis by differentially affecting tumor angiogenesis. Oncogene 29, 1351–1361, doi: 10.1038/onc.2009.426 (2010). 20010874

[b58] Lacerte A. . Transforming growth factor-beta inhibits telomerase through SMAD3 and E2F transcription factors. Cell Signal 20, 50–59, doi: 10.1016/j.cellsig.2007.08.012 (2008). 17881189

[b59] Rosas C., Sinning M., Ferreira A., Fuenzalida M. & Lemus D. Celecoxib decreases growth and angiogenesis and promotes apoptosis in a tumor cell line resistant to chemotherapy. Biological research 47, 27, doi: 10.1186/0717-6287-47-27 (2014). 25027008 PMC4101715

[b60] Bocca C., Bozzo F., Bassignana A. & Miglietta A. Antiproliferative effects of COX-2 inhibitor celecoxib on human breast cancer cell lines. Molecular and cellular biochemistry 350, 59–70, doi: 10.1007/s11010-010-0682-4 (2011). 21140284

[b61] Brandao R. D. . A randomised controlled phase II trial of pre-operative celecoxib treatment reveals anti-tumour transcriptional response in primary breast cancer. Breast cancer research : BCR 15, R29, doi: 10.1186/bcr3409 (2013). 23566419 PMC3672758

[b62] Chow L. W., Wong J. L. & Toi M. Celecoxib anti-aromatase neoadjuvant (CAAN) trial for locally advanced breast cancer: preliminary report. The Journal of steroid biochemistry and molecular biology 86, 443–447 (2003). 14623542 10.1016/s0960-0760(03)00355-8

[b63] Chow L. W., Yip A. Y., Loo W. T., Lam C. K. & Toi M. Celecoxib anti-aromatase neoadjuvant (CAAN) trial for locally advanced breast cancer. The Journal of steroid biochemistry and molecular biology 111, 13–17, doi: 10.1016/j.jsbmb.2008.04.004 (2008). 18514508

[b64] Falandry C., Canney P. A., Freyer G. & Dirix L. Y. Role of combination therapy with aromatase and cyclooxygenase-2 inhibitors in patients with metastatic breast cancer. Annals of oncology : official journal of the European Society for Medical Oncology/ESMO 20, 615–620, doi: 10.1093/annonc/mdn693 (2009). 19254941

