# Supplementary material for: Cyclooxygenase-2 regulates TGFβ-induced cancer stemness in triple-negative breast cancer
Source: Sci Rep. 2017 Jan 5;7:40258. doi: 10.1038/srep40258 (PMC5215509; doi:10.1038/srep40258)
Supplement: Supplementary Information [file srep40258-s1.pdf]

Cyclooxygenase-2 regulates TGFβ-induced cancer stemness in triple-negative breast cancer

Jun Tian[1], Mahmood Y. Hachim[1], Ibrahim Y. Hachim[1], Meiou Dai[1], Chieh Lo[1], Fatmah Al Raffa[1], Suhad Ali[1], Jean Jacques Lebrun[1]*

Supplementary Figure Legends

Figure S1: COX-2 expression in different molecular subtypes and its association with patient outcome.

a, Box plot of COX-2 gene expression in different breast cancer subtypes according to RSCMC classification using Breast Cancer Gene-Expression Miner v4.0. **b,** Box plot of COX-2 gene expression in different breast cancer subtypes according to Hu classification using Breast Cancer Gene-Expression Miner v4.0. **c,** Box plot of COX-2 gene expression in different breast cancer subtypes according to PAM50 classification using Breast Cancer Gene-Expression Miner v4.0. **d and e,** Kaplan-Meier survival analysis showing the relationship between COX-2 expression and 5-year OS outcome and 5-year DMFS outcome as well as AE free survival in patients who have luminal A and luminal B tumors using Kaplan Meier-plotter database and Breast Cancer Gene-Expression Miner v4.0. The classification methods of tumor subtypes are indicated.

Figure S2: Effects of TGFβ on cell growth and expression of mesenchymal and luminal markers in SUM159 and SUM149.

a, SUM159 and SUM149 cells were stimulated with TGFβ for 48 hours and subjected to MTT assay. **b,** Total RNA was extracted from adherent cells and P1 tumorspheres in the presence or absence of TGFβ. Indicated gene expression were measured by real-time qPCR in SUM159 and SUM149 cells.

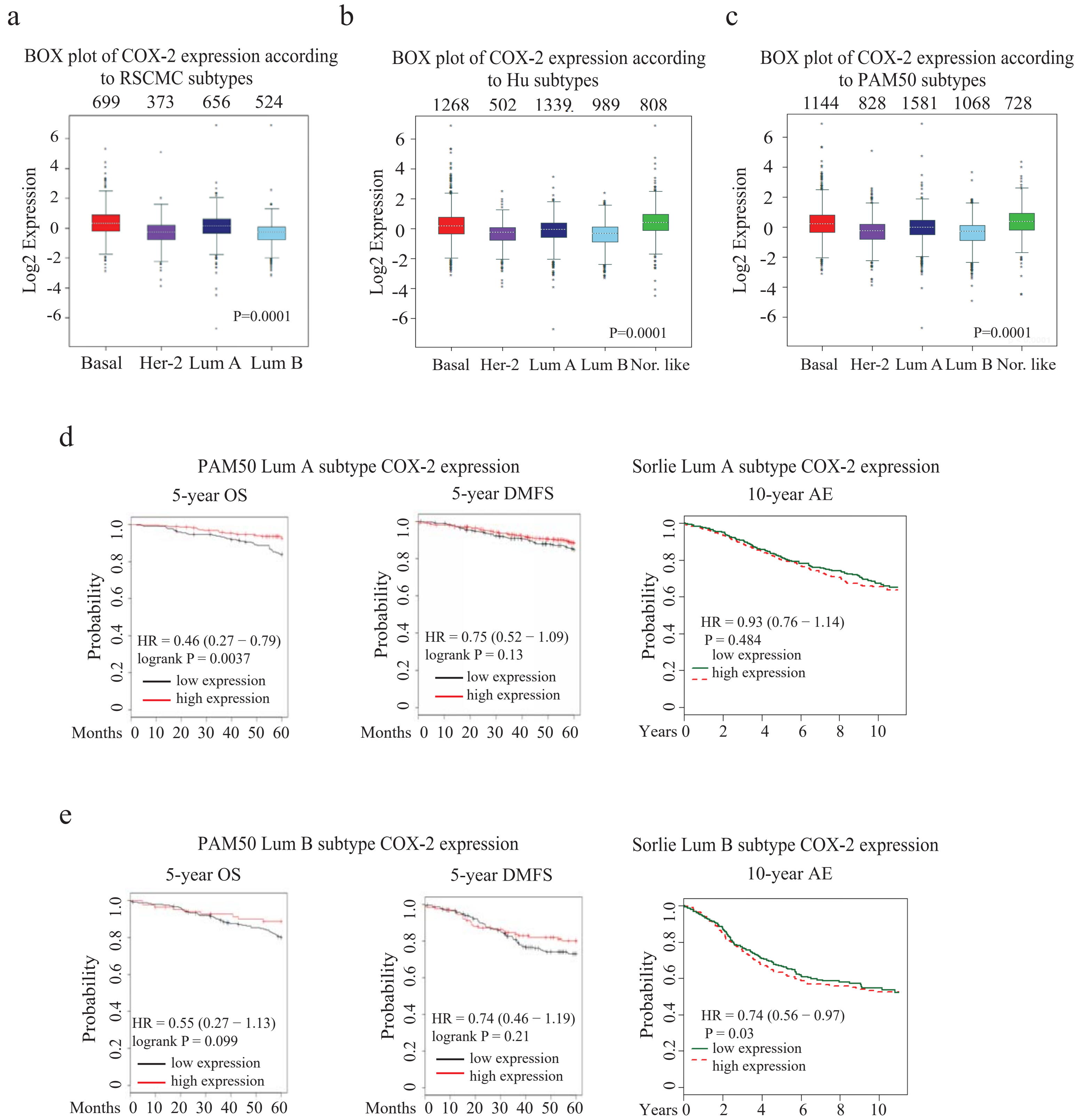
a
BOX plot of COX-2 expression according to RSCMC subtypes
699 373 656 524
Log2 Expression
P=0.0001
Basal Her-2 Lum A Lum B
b
BOX plot of COX-2 expression according to Hu subtypes
1268 502 1339 989 808
Log2 Expression
P=0.0001
Basal Her-2 Lum A Lum B Nor. like
c
BOX plot of COX-2 expression according to PAM50 subtypes
1144 828 1581 1068 728
Log2 Expression
P=0.0001
Basal Her-2 Lum A Lum B Nor. like
d
PAM50 Lum A subtype COX-2 expression
5-year OS
Probability
HR = 0.46 (0.27 − 0.79)
logrank P = 0.0037
low expression
high expression
Months
5-year DMFS
HR = 0.75 (0.52 − 1.09)
logrank P = 0.13
low expression
high expression
Months
Sorlie Lum A subtype COX-2 expression
10-year AE
HR = 0.93 (0.76 − 1.14)
P = 0.484
low expression
high expression
Years
e
PAM50 Lum B subtype COX-2 expression
5-year OS
Probability
HR = 0.55 (0.27 − 1.13)
logrank P = 0.099
low expression
high expression
Months
5-year DMFS
HR = 0.74 (0.46 − 1.19)
logrank P = 0.21
low expression
high expression
Months
Sorlie Lum B subtype COX-2 expression
10-year AE
HR = 0.74 (0.56 − 0.97)
P = 0.03
low expression
high expression
Years

a

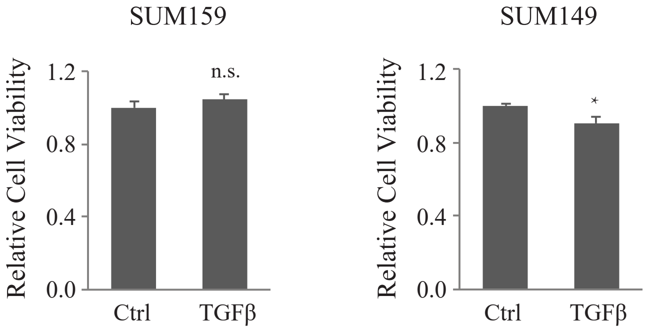
SUM159
SUM149
Relative Cell Viability
1.2
0.8
0.4
0.0
n.s.
*
Ctrl
TGFβ

b

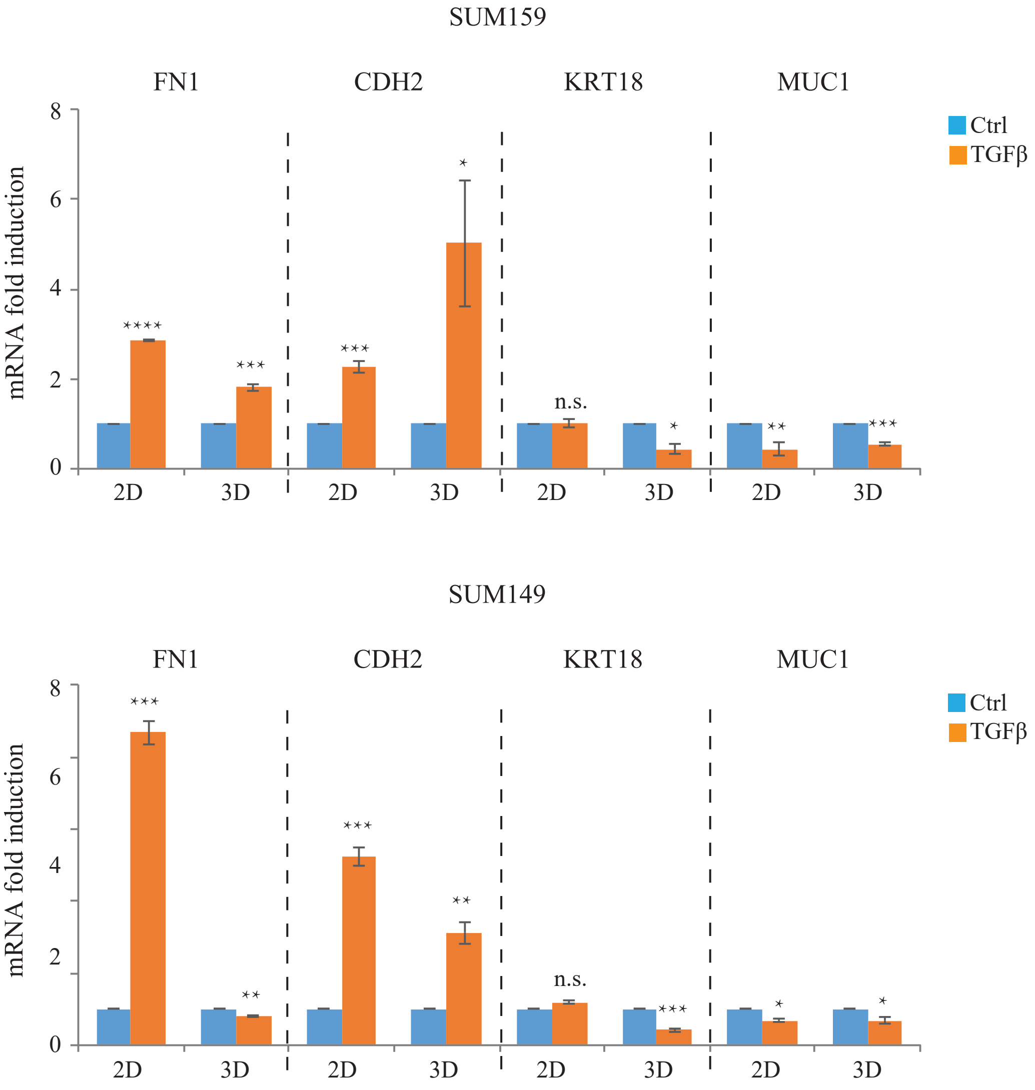
SUM159
SUM149
FN1
CDH2
KRT18
MUC1
mRNA fold induction
8
6
4
2
0
Ctrl
TGFβ
2D
3D
****
***
**
*
n.s.
